# The application of 3D printing in preoperative planning for transcatheter aortic valve replacement: a systematic review

**DOI:** 10.1186/s12938-022-01029-z

**Published:** 2022-09-01

**Authors:** Paris Xenofontos, Reza Zamani, Mohammad Akrami

**Affiliations:** 1 Medical School, College of Medicine and Health, Exeter, UK; 2 Department of Engineering, College of Engineering, Mathematics, and Physical Sciences, University of Exeter, Exeter, UK

**Keywords:** Additive Manufacturing, Rapid Prototyping, Transcatheter aortic valve implantation, TAVI, SAVR

## Abstract

**Background:**

Recently, transcatheter aortic valve replacement (TAVR) has been suggested as a less invasive treatment compared to surgical aortic valve replacement, for patients with severe aortic stenosis. Despite the attention, persisting evidence suggests that several procedural complications are more prevalent with the transcatheter approach. Consequently, a systematic review was undertaken to evaluate the application of three-dimensional (3D) printing in preoperative planning for TAVR, as a means of predicting and subsequently, reducing the incidence of adverse events.

**Methods:**

MEDLINE, Web of Science and Embase were searched to identify studies that utilised patient-specific 3D printed models to predict or mitigate the risk of procedural complications.

**Results:**

13 of 219 papers met the inclusion criteria of this review. The eligible studies have shown that 3D printing has most commonly been used to predict the occurrence and severity of paravalvular regurgitation, with relatively high accuracy. Studies have also explored the usefulness of 3D printed anatomical models in reducing the incidence of coronary artery obstruction, new-onset conduction disturbance and aortic annular rapture.

**Conclusion:**

Patient-specific 3D models can be used in pre-procedural planning for challenging cases, to help deliver personalised treatment. However, the application of 3D printing is not recommended for routine clinical practice, due to practicality issues.

## Introduction

Aortic stenosis (AS) affects between 3 and 7% of the population above the age of 65, which makes it the most common valvular heart disease in the developed world [1, 2]. AS is characterised as progressive hardening and narrowing of the aortic valve [3]. Severe stenosis is associated with significantly low survival rates at two years following symptom onset [4, 5]. Replacement of severely stenotic valve is therefore necessary to reduce morbidity and mortality associated with AS [6].

The currently available treatment options for symptomatic, severe AS are surgical aortic valve replacement (SAVR) and transcatheter aortic valve replacement (TAVR) [7, 8]. Since its introduction in 2002, TAVR has been established as the preferred treatment option for severe, progressive AS in patients with prohibitive surgical risk [9, 10]. TAVR, also known as transcatheter aortic valve implantation (TAVI), is a method by which a self- or balloon-expandable (S-E/B-E) bioprosthetic valve is delivered at the designated location by a catheter that is advanced into the vasculature through a peripheral artery [11]. The implanted valve displaces the leaflets of the existing calcified and stenotic valve to restore normal blood flow. Every year, around 3,250 people undergo TAVR in the UK [12]. New research findings have shown that TAVR is a non-inferior, or even superior treatment option compared to SAVR for low-surgical risk groups [13, 14]. The number of patients treated with TAVI is therefore expected to increase within the next few years.

TAVR has received great attention for being less invasive compared to open-heart SAVR, with significant reduction in risk of strokes, major bleeding and atrial fibrillation [15, 16]. Despite this, several complications are more prevalent in TAVR. These include paravalvular leak (PVL), new-onset conduction disturbance (NOCD) requiring permanent pacemaker implantation (PPI), rupture of the aortic annulus and coronary artery obstruction (CAO). PVL refers to the retrograde flow of blood from the aortic root into the left ventricle [17]. Failure to achieve circumferential seal between the bioprosthetic valve and the aortic annulus may result in regurgitation [18]. Conduction disturbances arise due to high pressures exerted by the valve frame on the critical region of the heart, where the conduction pathways are located [19]. Given that there is a large variety of transcatheter aortic valve (TAV) sizes and designs to choose from, understanding the patient’s anatomy is crucial in selecting the valve that obtains the best fit. Pre-procedural imaging scans provide vital information about the anatomy of the patient’s aortic root, which help clinicians decide which TAV system and implantation depth to adopt on a case by case basis [20, 21]. Procedural planning involves a multidisciplinary team approach with input from radiologists, clinical and interventional cardiologists [22]. Even with thorough planning, the absence of a standardised strategy to predict how the prosthetic valve will adapt in situ means that certain complications are extremely difficult to anticipate.

Advancements in the field of three-dimensional (3D) printing have made it possible to obtain from pre-procedural imaging scans of patients a physical replica of the individuals’ unique anatomy. The 2D volumetric data provided by cardiac Computed Tomography (CT), Magnetic Resonance Imaging (MRI) or echocardiography can be converted into patient-specific 3D models by means of additive manufacturing [23, 24]. In fact, 3D printed models have been used extensively in the field of cardiology to educate medical students and train surgical or interventional trainee doctors [25–27]. Patient-specific 3D printed models have also been used to aid in the doctor–patient communication and improve the process of informed consent [28].

More recently, patient-specific anatomic models have been used in the field of valvular heart disease. The physical replicas enable physicians to simulate the procedure by inserting the bioprosthetic valve in the models. Subsequently, the interaction between the patient’s anatomy and the valve can be assessed. Certain anatomical features, such as the volume of aortic annulus calcifications or the ovality of the valve’s landing zone, are factors that are known to affect the compatibility of the currently available valves with the patient’s anatomy [29]. Having discussed that models have the potential to represent the anatomical characteristics of each patient, simulation of TAVR on 3D printed models may be useful for preoperative planning. It could provide information on the clinical outcomes and on the risk of occurrence of postoperative complications. If this is true, then 3D printing could be used to address some of the current challenges of the procedure, such as the selection of suitable patients to undergo TAVR or the selection of the most appropriate valve for each case.

Due to the novelty of the application of 3D printing as a pre-surgical planning tool for TAVR, it remains unclear whether 3D models can be used to accurately predict the occurrence of intra- or post-procedural complications (e.g. PVL, CAO, NOCD etc.). The usefulness of simulating the TAVI procedure on patient-specific 3D printed models, with the aim to minimise complication severity or risk of occurrence, remains to be established. Previous systematic reviews have described the application of 3D printing in cardiovascular surgery and interventional radiology, but none has evaluated its use in TAVR [30, 31].

The aim of this systematic review is to evaluate the application of patient-specific 3D printed models in preoperative planning for TAVR. The objectives are to (a) to understand the accuracy of predicting TAVR associated complications through the use of 3D printed models, (b) to appreciate whether pre-surgical planning using these models can moderate the risk of occurrence of adverse events and (c) to understand the practicality and usefulness of 3D models in clinical practice.

## Results

### Study selection

**Fig. 1:**
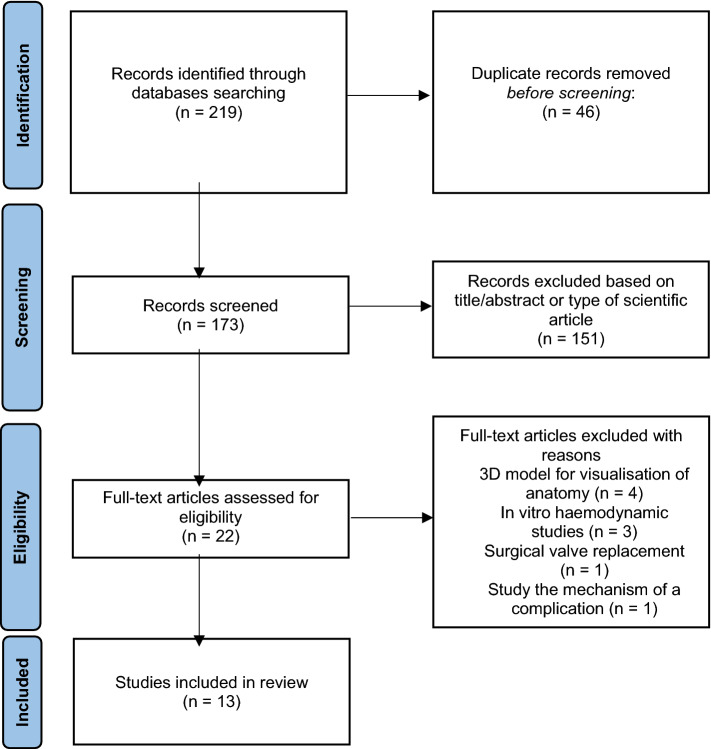
PRISMA flow chart. The PRISMA diagram was adapted from Moher D, Liberati A, Tetzlaff J, Altman DG, The PRISMA Group (2009) [32]

As shown by the PRISMA figure (Fig. 1), the literature search yielded a total of 219 records. Duplicate articles were excluded narrowing the results down to 173. Articles were then screened against the eligibility criteria on the basis of title, abstract and type of scientific article which further narrowed the results down to 22. The full text of the remaining articles was retrieved and nine studies were excluded. The most common reason for exclusion was the use of 3D models to simply visualise the patient’s anatomy, without application of model in TAVR planning or for prediction of complications. The use of models for the characterisation of haemodynamic changes post-valve deployment was the second most common reason for exclusion. One study was excluded because it had utilised 3D models to test their assumption for the underlying mechanism of a complication and another study because it had utilised 3D models for benchtop prediction of PVL following SAVR. A total of 13 papers were left for inclusion in this systematic review.

### Study characteristics and demographics

The study characteristics, patient demographics, postoperative clinical outcomes and complications of TAVR are summarised in Table 1. PVL is the most commonly studied complication. Six studies (6/13) have retrospectively recruited between five and 30 patients that had undergone TAVR [32–37]. They explored whether patient-specific 3D printed models could be used to predict the occurrence, location and/or severity of PVL. Three papers (3/13) performed TAVR on the 3D models of a sum of four patients, who had intra-procedural CAO [38–40]. The aim was to explore whether simulation of the procedure, using the same implantation technique as in the clinical setting, could predict the occurrence of the adverse event. Hatoum et al. tested a range of TAV sizes, designs and implantation approaches to identify which method could have been used in the actual procedure to prevent CAO [40]. Zhang et al. investigated the possibility of predicting intra-procedural aortic annular rupture by performing the surgery on the models of two patients who died as a result of this complication [38]. One study (1/13) utilised the pre-surgical imaging data of a patient who had experienced NOCD following TAVR, to 3D print a patient-specific model [41]. They tested a range of valve sizes and implantation depths, to (a) observe if they could use the model to predict the in vivo outcomes and (b) to understand which TAV approach could have prevented the adverse event. In two studies (2/13) the anatomies of two challenging cases, with high intra-procedural complication risk, were reconstructed using 3D printing [42, 43]. TAVI was performed inside the physical models to prospectively assess the safety of TAVR. One study (1/13) tested a newly developed valve delivery method that can be used to achieve native and bioprosthetic valve commissural alignment [44]. They initially recruited 3 patients whose anatomies were replicated using 3D printing. The patient-specific models were used to test the efficacy of the newly developed approach in achieving commissural alignment, before performing the surgery in vivo. The total number of patients across all 13 studies included in the review is 107. Seven (7/13) studies do not report the severity of AS, while two (2/13) studies report the surgical risk score of their population.

**Table 1:** Study characteristics, patient demographics and postoperative clinical outcomes

Reference	Study design					Cardiovascular profile		TAVR intervention		Patient Outcomes / Complications
	Complication / Study aim(s)	Recruitment [Retrospective (R)/ Prospective (P)]	Number of participants	Age range	Gender (%): male(M) / female (F)	AS Severity	Surgical risk score	In vivo	In 3D model	
Schmauss et al., 2012 [39]	CAO	R	1	70	M: 100%	Severe—very severe	N/A	26-mm B-E Sapien	26-mm B-E Sapien	Death due to CAO
Ripley et al., 2016 [32]	PVL	R	16	69–91	F: 31% M: 69%	N/A	N/A	B-E Sapien / Sapien 3 Re-ballooning on 6 occasions	3D printed closed based valve, valve diameter same as in vivo	7 mild PVL, 2 Moderate PVL, 7 no PVL
Fujita et al., 2016 [43]	Risk of injury to prosthetic mitral valve	P	1	82	F: 100%	Severe	STS—7.6%	26-mm B-E Sapien XT, delivered via the same diameter guidewire as in vitro	Guidewire with small curve: 42 mm	Uneventful TAVI
Qian et al., 2017 [37]	PVL	R	18	56–95	F: 56% M: 44%	N/A	N/A	First and second-generation S-E CoreValve, Re-ballooning in 7 cases	Same as in vivo	6 none, 5 trace-to-mild, 6 moderate, 1 moderate-severe PVL. Post-ballooning reduced significant PVL in 3 cases
Hosny et al., 2018 [36]	Prediction of valve size used in vivo, PVL	R	30	71–92	F: 57% M: 43%	N/A	N/A	B-E Sapien or Sapien XT, S-E CoreValve or Core Evolut R, S-E St. Jude Portico	3D printed valve sizer based on Sapien XT specifications	15 with at least mild PVL and 15 with no PVL
Tanaka et al., 2018 [33]	PVL	R	6	68–88	F: 83% M: 17%	Severe	N/A	23-mm B-E Sapien XT, ad hoc post-dilation on 2 cases	23-mm B-E Sapien XT, filling volume of deployment balloon and ad hoc post-dilation as in vivo	3 mild, 1 mild-moderate, 1 moderate,1 moderate-severe PVL 1 died 1.3 years post-TAVR due to considerate amount of PVL due to undersized valve
Yaku et al., 2018 [42]	Safety of TAVR for a patient with high risk of injury to aorta	P	1	90	F: 100%	Severe	N/A	23-mm B-E Sapien 3	26-mm B-E Sapien 3, 29-mm S-E CoreValve Evolut R	Uneventful TAVR Post-op CT on day 7: no changes in the intramural haematoma. Patient doing well at 6 months
Hatoum et al., 2019 [40]	CAO	R	1	80	M: 100%	Severe—very severe	N/A	29-mm B-E Sapien 3	26, 29-mm B-E Sapien 3, 31-mm S-E CoreValve implanted in normal, supra- and sub-annular depth	Left CAO
Zhang et al., 2019 [38]	CAO and aortic annulus rapture	R	4	N/A	N/A	N/A	N/A	B-E Sapien XT	Non-valve stent model consistent with B-E Sapien XT size and radial force support specifications. Balloon valvuloplasty and balloon dilation performed as in vivo	2 died of CAO, 2 died of aortic annular rupture
Haghiashtiani et al., 2020 [41]	New-onset conduction disturbances	R	1	N/A	N/A	N/A	N/A	29-mm S-E CoreValve Evolut R at intermediate height	29-mm S-E CoreValve Evolut R at intermediate, shallow and deep height and 26-, 29-, 31-mm S-E Evolut R at intermediate height	One patient with new-onset conduction disturbance
Reiff et al., 2020 [35]	PVL	R	20	74–84	F: 30% M: 70%	N/A	STS—6.6%	23, 26-mm B-E Sapien XT	Same Implantation depth and valve type as in vivo. Nominal volume for balloon expansion	10 no PVL, 9 mild, and 1 moderate PVL
Thorburn et al., 2020 [34]	PVL	R	5	68–87	F: 20% M: 80%	N/A	N/A	B-E Sapien 3	Same valve type, size and implantation depth as in vivo	All patients had either none or trivial PVL
Redondo et al., 2021 [44]	Alignment of native and TAV commissures	P	3	N/A	N/A	Severe	N/A	S-E ACURATE neo valve as in vitro	S-E ACURATE neo valve	No commissural misalignment or coronary ostia obstruction reported

*STS* Society of Thoracic Surgeons

### Model construction and key characteristics

Table 2 summarises the types of 3D printers and materials used to construct the models. All (13/13) studies used the pre-procedural CT imaging data to reproduced the patients’ anatomies. Stereolithography (SLA) was the most commonly used 3D printing technique followed by material jetting. Time and/or costs of model construction are reported in four papers. Key model characteristics are also summarised in Table 2.

**Table 2:** 3D printed model construction and key characteristics

Reference	Model construction		3D printer	Material	Time	Cost	Model characteristics
	Imaging	Anatomy					
Schmauss et al., 2012 [39]	Cardiac CT	Aortic root, aortic arch and the ascending aorta	Polyjet	N/A	N/A	N/A	N/A
Ripley et al., 2016 [32]	ECG gated Cardiac CT, images at peak systole	Aortic root and LVOT. Valve leaflets not included	SLA	Clear flexible photosensitive resin	5 h	N/A	Agreement of minimum and maximum annulus diameter measurements between 3D model and patient's imaging data
Fujita et al., 2016 [43]	CT	Ascending aorta, aortic valve, prosthetic mitral valve and LVOT	SLA	Photosensitive resin	N/A	N/A	N/A
Qian et al., 2017 [37]	Contrast-enhanced CT, images taken at systolic phase	Aortic root, aortic annulus, LVOT and valve leaflets	Polyjet	Photopolymers: Stiff sinusoidal fibres—VeroBlackPlus® (RGD875) Elastic matrix—TangoPlus® (FullCure 930)	Segmentation of anatomical structures: 5–10 min Formation of digital files for printing: 5 min 9–10 h to print ten 3D models Post-printing processing time: 45 min	Cost of printing materials per model: $150 to $200	Model imitates, to some degree, the strain-stiffening characteristic of human soft tissue Model submerged in water at 37 °C to mimic temperature of body, ensured full expansion of the valve
Hosny et al., 2018 [36]	ECG gated cardiac CTA, images taken at diastolic phase	Aortic root, annulus, LVOT, valve leaflets with calcifications	Polyjet	Photopolymers: Calcified leaflets and valve sizer printed with rigid white VeroWhitePlus (RGD835), aortic root/ non-calcified leaflets printed with flexible transparent TangoPlus® (FLX930)	N/A	N/A	Agreement in annulus diameter measurements between 3D model and patient's imaging data Mechanical properties of human tissue were approximated but, strain-stiffening behaviour of human aortic tissue not replicated
Tanaka et al., 2018 [33]	ECG gated multi-detector CT, images taken at end-diastole	aortic annulus with valve leaflets, aortic root, LVOT, thoracic and abdominal aorta, iliofemoral arteries	SLA—for all structures except aortic annulus	Printed material not specified. Aortic annulus with three leaflets constructed using silicone moulding	N/A	N/A	Elasticity of calcified regions and mechanical stiffness of aortic annulus were adjusted to those of human tissue Pulsatile flow circulation system replicated HR and mAP of patients
Yaku et al., 2018 [42]	CT	N/A	SLA	Photosensitive resin: Aortic wall printed with Polyurethane resin. Intramural haematoma was made using epoxy resin (hard material)	N/A	N/A	Pressure gauge measure pressure exerted onto aortic wall
Hatoum et al., 2019 [40]	Cardiac CT, image taken at diastole	LVOT, aortic annulus with valve leaflets, aortic root and ascending aorta	Polyjet	Photopolymers: Calcified leaflets printed with rigid white VeroWhitePlus (RGD835), soft tissues printed with flexible transparent TangoPlus® (FLX930)	N/A	N/A	Model connected to pulse duplicator left heart simulator that replicated mAP and HR
Zhang et al., 2019 [38]	ECG gated cardiac CTA, images at systolic phase	Aortic root, valve leaflets with calcifications and LVOT	N/A	HeartPrint® Flex for non-calcified regions: transparent, flexible, mimicking modulus of elasticity of human arterial tissue Material for hard calcifications—N/A	N/A	N/A	Calcifications printed with a different coloured material to allow visualisation
Haghiashtiani et al., 2020 [41]	Cardiac CT	Aortic wall, aortic annulus, valve leaflets with calcifications, LVOT	Custom-built 3D printing system (AGS1000, Aerotech)	Silicone sealant and silicone grease mixed at various specified weight ratios to print (a) Myocardium and leaflets and (b) aortic wall. Speckling material for calcifications on vales. Colouring agent marked the intermediate implantation depth	3D models left in ambient air for 5 to 7 days after printing, to complete curing	N/A	Young's modulus of meta-materials fall within the range of moduli values for human tissue. Materials failed to represent strain-stiffening behaviour of human tissue at high strains
Reiff et al., 2020 [35]	ECG gated CT, images at systolic phase	LVOT, aortic root and ascending aorta Native leaflets not included	FDM	Thermoplastic polyurethane (Ninjaflex flexible)	N/A	N/A	Model approximates the modulus of elasticity of the human aorta
Thorburn et al., 2020 [34]	ECG gated cardiac CT	Aortic root, the coronary artery ostia and LVOT Native leaflets not included	FDM	Thermoplastic polyurethane (Ninjaflex flexible) Sealant material	Printing time alone: 4 h / model	N/A	Closed pressure system. Saline to represent blood. Radiopaque marker on the annulus to allow them to replicate implantation depth as in vivo
Redondo et al., 2021 [44]	ECG gated cardiac CT	Thoracic aorta, aortic arch, descending aorta, aortic root and coronary ostia	SLA	Photosensitive resin with flexible silicone-like characteristics	N/A	N/A	N/A

*CTA* Computed Tomography Angiography, *ECG* Electrocardiogram, *FDM *Fused Deposition Modelling, *HR* Heart Rate, *LVOT* Left ventricular outflow tract, *mAP* mean Arterial Pressure

### Study methodology and findings

#### Paravalvular leak

Ripley et al. developed a light transmission test that highlights the presence and location of gaps between the valve frame and the model’s aortic wall [32]. The severity of PVL was quantified by, the surface area of projected light as a percentage to the total annulus area. The sensitivity and specificity of their methodology for predicting the occurrence of PVL are 67% and 71%, respectively. Out of the six true positive cases, the predicted location of PVL was correct in five. The predicted severity for five patients with true mild PVL, ranged from 0.8 to 4.7%. For the single case of moderate PVL the predicted severity was 1.0%.

Tanaka et al. attached each 3D model with the implanted valve to a pulsatile flow circulation system that replicated the in vivo haemodynamic conditions [33]. Next, an electromagnetic flow sensor was used to measure the regurgitant flow rate. The flow rates obtained from the six replicas ranged from 0.45 to 1.18 L/min. In each case, the derived value matched the patient’s aortic regurgitation (AR) grade that was obtained via clinical echocardiography. In vivo AR grades ranged from mild to moderate-severe. Micro-CT was performed to measure the gap area between the wall of the model’s aortic annulus and the stent frame. In five (5/6) cases, the surface area of the gap was consistent with each patient’s AR grade. In one (1/6) case the gap area predicted a larger PVL grade than clinically observed PVL. In five (5/6) cases, the location of the gap area in the model matched the site of the clinically observed leak. Similarly, Thorburn et al. developed a closed pressure system to quantitatively assess PVL [34]. Once the pressure difference across the valve reached 60 mmHg, the volume of regurgitant fluid (ml) was measured over 5 s. The process was repeated three times and the average rate of PVL (ml/s) was calculated for each model. The average rates obtained ranged from 19.1 to 24.1 ml/s. For each case, the rate of PVL was significantly associated with the degree of clinically confirmed leakage (*p* < 0.001).

Reiff et al. performed micro-CT on the 3D models following valve deployment, to visually examine for the presence and location of gap areas [35]. PVL severity was calculated as a percentage of the total circumference. Predicted outcomes were compared with each patient’s post-procedural PVL grade and location. The observer correctly predicted the absence of PVL in nine (9/10) cases and the presence of PVL in eight (8/10) cases. Six (6/9) patients with clinically confirmed mild PVL were classified as moderate. The predicted location of PVL was correct in eight (8/10) patients. The sensitivity and specificity of annular calcium volume (measured on pre-procedural CT) in predicting the occurrence of PVL were 60% and 90%, respectively. The sensitivity and specificity of annulus eccentricity index (AEI) were found to be 40% and 50%, respectively.

Hosny et al. designed a valve sizer that was inserted in each model and was sequentially opened to 20, 23, 26 and 29 mm that represent the range of diameters of the currently available valves [36]. The valve size, with a gap area that could not be further reduced by a greater size, was defined as "best fit". The observer correctly predicted the valve size that was used in vivo in 19 (64%) cases. In the six patients who received B-E valves and there was discrepancy between the predicted and actual valve size, the observer always predicted a larger size. Of those six patients, five (5/6) had clinical diagnosis of PVL. Prediction of PVL occurrence was decided on visual confirmation of gap areas. The sensitivity and specificity of the method used to predict PVL were 60% and 73%, respectively.

To quantify post-TAVR annular strain, Qian et al. inserted radiopaque beads to their models [37]. They measured the displacement of the beads by performing a CT scan before and after TAV deployment. Areas of focal strain unevenness were determined by calculating a bulge index. The sensitivity and specificity of the maximum bulge index for predicting significant PVL were 71% and 82%, respectively. The best predictor of significant PVL was the volume of annular calcium (mm^3^) measured on pre-TAVR CT, with sensitivity and specificity of 86% and 72%, respectively. Annular ellipticity was a poor predictor of PVL. Bulge index was the only predictor of PVL following ad hoc post-dilation. The location of maximum bulge index matched the dominant PVL site in nine (9/12) patients.

#### New-onset conduction disturbances

Haghiashtiani et al. inserted pressure sensors in the walls of a patient-specific 3D model, at the site where the conduction pathways of the heart are located [41]. A range of valve sizes and implantation depths were tested. Heatmaps allowed to visually assess the pressure exerted by each valve on the critical region. The maximum pressure values yielded by the 29-mm valve implanted at a shallow, intermediate and deep height were 234, 486 and 404 kPa, respectively. The pressure values for the 26-, 29- and 31-mm valves implanted at intermediate height were 60, 375 and 528 kPa, respectively.

#### Coronary artery obstruction and aortic annular rapture

Zhang et al. observed the outcomes of valve deployment in four patient-specific 3D models by means of endoscopy [38]. The displaced valvular calcifications in one case, and the distal edge of the valve frame in the second model, obstructed the left coronary ostia of the 3D models. In the other two patient-specific replicas, expansion of the B-E valves resulted in rapture of the aortic annulus. In vitro simulations replicated the in vivo complications of each case. In another study, Schmauss et al. printed the anatomy of a patient who died as a result of intra-procedural CAO [39]. Based on observations during the valve implantation in the model, it was concluded that the small and non-compliant sinuses of Valsalva may have necessitated a deeper implantation of the valve frame in the aortic annulus. This could avoid occlusion of both coronary ostia. Hatoum et al. attached the model of a patient who experienced CAO during TAVR, to a left heart pulse simulator [40]. The patient’s preoperative haemodynamics were replicated. The coronary artery flow rate was measured before and after valve implantation. Coronary obstruction was quantified using the Fractional Flow Reserve (FFR) equation, which is the rate of coronary blood flow post-TAVR as a percentage to the pre-procedural coronary blood flow rate. FFR below 75% was defined as inadequate coronary perfusion. The FFR of the 29-mm B-E Sapien 3 was 45.7 ± 0.6%, while the 26-mm B-E Sapien 3 expanded with a 29-mm balloon gave an FFR of 92.1 ± 1.2%. The 31-mm S-E CoreValve in the supra-annular and sub-annular implantation depths yielded FFR values of 89.6 ± 1.1% and 98.3 ± 1.1%, respectively.

Redondo et al. obtained the preoperative CT scans of three patients to construct their anatomy on a computer software [44]. TAVI was simulated in silico, in order to estimate the degrees of required rotation needed to obtain commissural alignment of native and prosthetic valves. TAVs were deployed in the 3D models according to the calculated patient-specific rotation, as estimated in silico. No coronary ostia overlap was detected in any of the models. TAVI was then performed on 3 patients according to the specific rotation that was calculated in silico and tested on the 3D models. Postoperative CT scans confirmed the absence of coronary ostia obstruction.

#### Challenging cases

Yaku et al. identified an aortic intramural haematoma on the pre-procedural CT scan of a patient, whose anatomy was replicated using 3D printing [42]. A pressure gauge was used to measure the force exerted on the haematoma during the advancement of the catheter in the model. The maximum pressures exerted from the S-E and B-E valve catheters were 155 ± 41 mmHg and 120 ± 14 mmHg, respectively. The B-E valve was chosen to be deployed in vivo. In another case, the prosthetic mitral valve was shown to be very close to the aortic annulus [43]. Concerns were raised as the interaction of the TAVR guidewire with the mitral valve could have caused irreversible valve dysfunction. TAVR was performed on the patient-specific anatomic model with the selected guidewire being safely inserted in the left ventricle, without interference with the mitral valve. The same guidewire was used for the clinical procedure.

## Discussion

According to the results of this systematic review, simulation of TAVR on patient-specific 3D printed models portray to be an accurate way of predicting post-procedural occurrence of PVL. The models could be used as pre-surgical planning tools in challenging cases, enabling the delivery of personalised TAVI treatment for better outcomes. There is evidence to support that in the future, 3D printed anatomical replicas could be used to reduce the incidence of procedural CAO, aortic annular rapture and lower the proportion of patients that require PPI following TAVR.

### Prediction of TAVR complications

In the 13 studies included, 3D printing was mostly applied to predict paravalvular regurgitation, of which six papers assessed this complication [32–37]. The review highlights that patient-specific 3D printed models can be used in various ways to predict post-procedural occurrence of PVL. The findings show that each methodology has a different accuracy in correctly identifying true positive and true negative cases. The light transmission test developed by Ripley et al., was more sensitive in predicting the occurrence of PVL, compared to the methodology adopted by Hosny et al. [32, 36]. The latter created a valve sizer according to B-E Sapien valve specifications, which was used to simulate TAVI in the physical models. Of note, S-E valves have different designs and dimensions to B-E valves. Given that half of the recruited patients received S-E valves, using the valve sizer to retrospectively predict the incidence of PVL may have compromised the sensitivity of their test. Reiff et al. have found that patient-specific 3D models with implanted valves can be used to predict the presence of paravalvular leak with high sensitivity (80%) and specificity (90%) [35]. Similarly, Qian et al. have shown that maximum bulge index was the second-best predictor of PVL, demonstrating that 3D models can be used to predict paravalvular AR with fairly high sensitivity and specificity [37]. Although to this day, no single risk factor has been found to be a perfect predictor, several anatomical characteristics have been shown to be associated with PVL. Annular calcium volume score > 3000 AU is considered the most significant anatomical predictor of PVL, with sensitivity of 86% and specificity of 80% [45]. The AEI is used to define the ovality of the aortic annulus, with zero indicating a perfect circle [46]. Wong et al. have shown that an AEI of greater than 0.25 can predict the occurrence of PVL with a sensitivity and specificity of 80% and 86%, respectively [46]. It appears that simulation of TAVR on patient-specific 3D printed models can achieve comparable sensitivity values to the most significant anatomical predictors of PVL. However, the results should be interpreted with caution. The studies have retrospectively recruited their participants, and as such knew which patients had clinically confirmed leakage. This may have introduced bias during the in vitro assessment of PVL, favouring higher sensitivity and specificity values. Before making any recommendations for the application of 3D printing in clinical practice, future studies should aim to use 3D models to predict the occurrence of PVL prospectively.

A number of studies have found that even mild PVL is associated with significantly poorer long-term outcomes and higher mortality rates [47, 48]. Furthermore, post-implantation procedures, such as balloon post-dilation, as a means of reducing the severity of PVL, carry additional risks [49]. Emerging data highlight the necessity for accurate prediction of PVL severity preoperative, in order to carefully select the size and type of valve to be implanted. Predicting the severity of PVL outside the human body can be particularly challenging, due to the fact that regurgitant volume is influenced by haemodynamic conditions and tends to be greater during systole [50]. This might explain why eligible studies that tried to predict the grade of PVL without taking into consideration the dynamic nature of paravalvular AR across the cardiac cycle have failed to get accurate predictions [32, 35, 37]. This review supports that imitating the in vivo haemodynamic conditions inside 3D models that resemble in situ tissue with realistic tensile and texture strength and flexibility could be an accurate way of predicting the severity of PVL. However, unless further studies with larger sample size reproduce these findings, the clinical application of 3D printing for the prediction of PVL severity cannot be recommended yet.

Oversizing of the TAV relative to the circumference of the aortic annulus by more than 25% may cause the annulus to rapture due to exertion of high radial force [51]. One study has shown that practising TAVR on 3D models could be useful in predicting aortic annular rapture [38]. To predict this complication, the materials used to 3D print the models should approximate the modulus of elasticity of human aortic tissue. Currently available 3D printing materials, exhibit strain-softening mechanical behaviour when subjected to tension, which is opposite to the strain-stiffening behaviour of normal human vasculature [33, 35, 36, 38, 41]. Wang et al. have demonstrated that multi-material 3D printers can be used to create meta-materials with similar mechanical properties to human tissue [52]. This was achieved by embedding sinusoidal wave structures, printed using a stiff material, to the soft wall of the 3D models. In this way, they were able to approximate the strain-stiffening properties of human tissue, and have shown that it is possible to print patient-specific tissue-mimicking 3D printed models. Future studies should replicate the anatomy of patients with postoperative aortic annular rapture, to explore the accuracy of using patient-specific tissue-mimicking 3D models to predict the adverse event.

### Safety of TAVR

This review supports that pre-surgical rehearsal of TAVI on patient-specific 3D printed models can help mitigate the risk of occurrence of certain procedural complications. In particular, Hatoum et al. tested different valve sizes, types and implantation depths, and concluded that a deeper implantation of an S-E valve could have prevented the occurrence of CAO [40]. This raises the issue as to whether pre-procedural benchtop TAV deployment in 3D models could have prevented the death of four patients who died as a result of CAO. It can be argued that preventative planning by means of 3D printing may help improve the safety of the procedure. To strengthen this argument, this review draws the reader’s attention to two studies that used 3D printing to ensure the safety of TAVR prospectively [42, 43]. The challenging anatomies of two patients were printed, enabling interventional cardiologists to practise different catheter advancement methods. In each case, the surgeons had selected the personalised approach that was shown to be associated with the highest chance of procedural success. The outcomes of the clinical procedures, which are summarised in Table 1, show that no adverse events had occurred during TAVR and that the patient with the aortic intramural haematoma was doing well 6 months postoperatively.

A proportion of patients who require PPI following TAVR remain undesirably high [53]. Haghiashtiani et al. have shown that 3D models with internal pressure sensors can be used to reduce the incidence of NOCDs [41]. Their tests have shown that, had the selected valve been implanted in a supra-annular position, the conduction disturbance might have been prevented. Shallow positioning of the 29-mm valve was found to exert lower pressure on the critical region and thus lower chance of disrupting the conduction of signals through the pathways. Simulation of TAVR on the 3D model could have predicted the occurrence of the adverse event and would possibly guide the selection of the appropriate depth of implantation. However, the pressure threshold values, above which conduction disturbances occur have not been previously established, which further complicates the process of preventative planning. This could be a topic for future research. Studies should recruit large cohorts of patients with and without post-procedural conduction disturbances and utilise patient-specific models with internal sensors to define threshold values. The 3D models could then be used in clinical practice, to guide the selection of the most appropriate valve size, type and implantation depth on an individual patient basis.

### Usefulness and practicality

This review aimed to evaluate the application of 3D printing for TAVR, in order to explore the possibility of introducing this technology as a standardised pre-surgical planning tool in clinical practice. As previously mentioned, pre-procedural imaging scans are useful in measuring certain anatomical features, which help clinicians plan for the procedure. However, physical interactions between the patient’s anatomy and the valve prosthesis cannot be inferred from imaging data. For instance, the valve tends to maintain circularity during expansion in situ and subsequently may reduce the ovality of the annulus [54]. These physical interactions can be replicated inside 3D models and may therefore provide better insight into the adaption of the valve after deployment [55]. Although 3D printing may help to prevent the occurrence of several complications, the practicality of its use in clinical practice remains unclear. Production of high-quality patient-specific models is both time consuming and costly [31]. Tertiary centres offering TAVI would need to be supplied with expensive, SLA or material jetting printers that will be able to print the patient’s anatomy with high accuracy. Furthermore, appropriately trained personnel would be required to segment the desired anatomy and prepare the digital files for printing. Pre-procedurally, surgeons would need to spend lots of time in testing several different valves which may be impractical in emergency situations. Transcatheter heart valves are expensive themselves, and testing a range of different types and sizes in each model may not be a sustainable option. Other TAVR complications, such as intra-procedural major bleeding or post-procedural stroke events, will be much more difficult to replicate and consequently plan for using 3D printing. Finally, if the clinically recommended prosthesis is tested on 3D models and found to obtain inappropriate fit, it is unclear how such disagreements should be resolved. Taking into consideration the findings of this review and the discussed limitations of 3D printing, there is currently weak evidence to support the application of 3D printed models as a standardised pre-surgical planning tool for TAVR. Large randomised controlled trials should examine the effects of preoperative planning using patient-specific models on appropriate clinical outcomes, used in the conduct of TAVR clinical research, such as all-cause mortality, length of hospitalisation, presence and severity of valve-related complications or patient-reported quality of life [56].

## Study limitations

Despite all efforts to select the most appropriate evidence for this review, it is possible certain bias would have been introduced in the rejection of research report, when considering eligibility. Despite this, the eligibility criteria are summarised in Table 1, which allow independent researchers to review the study selection process. Quality assessment of included studies was not performed due to the absence of a standardised assessment tool for proof-of-concept studies. This highlights the need for a standardised methodology to assess the risk of bias in feasibility studies.

## Conclusion

This is the first systematic review that aims to evaluate the application of 3D printing in pre-procedural planning for TAVR. The findings of this review show that patient-specific 3D printed models can be used to predict the occurrence and severity of PVL with reasonable accuracy. The existing evidence is limited by the small population size and retrospective nature of these proof-of-concept studies that could be addressed in future research. Prospective assessment would provide better insight into the sensitivity and specificity of benchtop TAVR simulation in predicting paravalvular regurgitation. For the prediction of aortic annular rapture, the review suggests that tissue-mimicking 3D models may be a better way forward in observing whether the annulus can withstand the tensile load of the valve frame. Future research is needed to explore the clinical usefulness of these models in mitigating the risk of aortic annular rapture. This review shows that it is feasible to use patient-specific 3D printed models to test a range of valves and implantation strategies, in order to deliver personalised treatment with lower risk of complication occurrence. Adverse events, such as CAO or cardiac arrhythmias, requiring the insertion of permanent pacemakers, could be prevented with the help of 3D printing. Due to the experimental nature of the studies on this topic, further research is required to produce clinically relevant evidence in order to draw more concrete conclusions. From a clinical perspective, 3D models can be used to complement the current clinical practices in planning for challenging cases. Despite this, due to practicality issues, patient-specific 3D printed models are not recommended for routine clinical practice, as a means of facilitating the decision-making for the delivery of individual patient care.

## Methods

### Eligibility criteria

Studies were eligible for inclusion in this review provided that the inclusion and exclusion criteria were met. The eligibility criteria are summarised in Table 3.

**Table 3:** Eligibility criteria

Inclusion criteria	Exclusion criteria
Participants with a diagnosis of aortic stenosis, who have undergone TAVR	The application of 3D printing for supravalvular aortic stenosis, aortic root replacement, minimally invasive aortic valve replacement or surgical aortic valve replacement
Use of preoperative imaging data from real patients, to segment cardiovascular region of interest and construct the models. This process allows the patient’s anatomy to be represented by the 3D object	Computational 3D modelling only
Application of any 3D printing method to either construct the patient’s anatomy or print a mould to cast 3D silicone parts	Studies that exclusively assess the feasibility of creating accurate and representative patient-specific 3D models from pre-procedural imaging data
Studies should (a) use patient-specific 3D models as pre-surgical planning tools to predict the occurrence of intra- or post-procedural TAVR associated complications (e.g. PVL, coronary artery obstruction, new-onset conduction disturbances etc.) or, (b) perform different TAV approaches (e.g. valve size, valve type, implantation depth, etc.) on models, with the aim to minimise the complication’s severity or risk of occurrence	Studies with a primary focus on using 3D models to imitate the haemodynamic changes after the deployment of the valve
The outcomes obtained through the simulation of TAVR on the 3D printed objects must be compared to the in vivo outcomes	Studies with a focus on exploring assumptions for the biophysical mechanism of complications following TAV placement
	Review articles, conference abstracts, editorial comments, letters and video–audio journals
	3D printed models for training cardiothoracic surgical trainees
	Studies with no access to full paper
	Articles not available in English language

### Information sources and search strategy

This review has followed the PRISMA guidelines for reporting systematic reviews [57]. The preferred scholar search engines were searched on the 28/01/2022 with no year limit: Web of Science, Embase and MEDLINE. Initially the search strategy was specific to 3D printing and TAVR associated complications, which gave very few results. The terms used to search for complications were replaced with universal terminology that refer to TAVR. This has helped expand on the results and make sure all relevant studies were identified. Relevant free text search terms to be identified in the title and/or abstract and Medical Subject Headings were combined using the Boolean AND operator. The full electronic search strategy of one of the databases is provided below.

### Study selection

The option to exclude review articles using the advanced search engine was not always possible, and therefore, in two databases the review papers were manually excluded. Following de-duplication, the title, abstract and type of scientific article were screened against the eligibility criteria by a single reviewer. The results were narrowed down to potentially relevant articles. Full-text screening of remaining papers was performed, whereby eligibility against the inclusion criteria was assessed.

### Data collection process

Data collection was performed by the reviewer and the following information was extracted from relevant papers: study design including study aims, number of participants, age range, gender, cardiovascular profile of patients (severity of AS and surgical risk score), TAVR approach used in vivo and on the 3D models, patient outcomes, study methodology and findings. Imaging scans used to construct the models, the anatomy represented by the models, type of 3D printer, materials used for model construction, time, costs and key model characteristics were also extracted if available.

## Data Availability

The datasets used and/or analysed during the current study are available from the corresponding author on reasonable request.

## Appendix A

Search Strategy:

1. TAVI.ti,ab.
2. TAVR.ti,ab.
3. (Transcatheter aortic adj2 replacement).ti,ab.
4. (Transcatheter aortic adj2 implantation).ti,ab.
5. Trans-catheter aortic valve replacement.ti,ab.
6. Trans-catheter aortic valve implantation.ti,ab.
7. aortic stenosis.ti,ab.
8. *Transcatheter Aortic Valve Replacement/
9. 3D print*.ti,ab.
10. 3-D print*.ti,ab.
11. Three dimensional print*.ti,ab.
12. Three-dimensional heart model.ti,ab.
13. 3-dimensional print*.ti,ab.
14. Printing, Three-Dimensional/
15. aortic valve model*.ab,ti.
16. 1 or 2 or 3 or 4 or 5 or 6 or 7 or 8
17. 9 or 10 or 11 or 12 or 13 or 14 or 15
18. 16 and 17
19. limit 18 to english language
20. limit 19 to "review articles"
21. 19 not 20

Full electronic search of MEDLINE database.

## Abbreviations

AEI: Annulus eccentricity index
AR: Aortic regurgitation
AS: Aortic stenosis
B-E: Balloon-expandable
CAO: Coronary artery obstruction
CTA: Computed Tomography Angiography
CT: Computed Tomography
ECG: Electrocardiogram
FDM: Fused Deposition Modelling
FFR: Fractional Flow Reserve
HR: Heart Rate
LVOT: Left ventricular outflow tract
mAP: Mean Arterial Pressure
MRI: Magnetic Resonance Imaging
NOCD: New-onset conduction disturbance
PPI: Permanent pacemaker implantation
PVL: Paravalvular leak
SAVR: Surgical aortic valve replacement
S-E: Self-expandable
SLA: Stereolithography
STS: Society of Thoracic Surgeons (score)
TAV: Transcatheter aortic valve
TAVI: Transcatheter aortic valve implantation
TAVR: Transcatheter aortic valve replacement
2D: Two-dimensional
3D: Three-dimensional
